# Roles of a Novel Molecule ‘Shati’ in the Development of Methamphetamine-Induced Dependence

**DOI:** 10.2174/157015911795017362

**Published:** 2011-03

**Authors:** Minae Niwa, Toshitaka Nabeshima

**Affiliations:** 1 Department of Chemical Pharmacology, Graduate School of Pharmaceutical Sciences, Meijo University, Nagoya 468-8503, Japan; 2 Department of Psychiatry, Nagoya University Graduate School of Medicine, Nagoya 466-8560, Japan; 3 Department of Psychiatry and Behavioral Sciences, Johns Hopkins University School of Medicine, Baltimore, MD 21287, USA; 4 The Academic Frontier Project for Private Universities, Comparative Cognitive Science Institutes, Meijo University, Nagoya 468-8503, Japan

**Keywords:** Shati, methamphetamine, dependence, tumor necrosis factor-α, dopamine, uptake, nucleus accumbens, anti-addictive.

## Abstract

The ability of drugs of abuse to cause dependence can be viewed as a form of neural plasticity. Recently, we have demonstrated that tumor necrosis factor-α (TNF-α) increases dopamine uptake and inhibits methamphetamine-induced dependence. Moreover, we have identified a novel molecule ‘shati’ in the nucleus accumbens of mice treated with methamphetamine using the PCR-select cDNA subtraction method and clarified that it is involved in the development of methamphetamine dependence: Treatment with the shati antisense oligonucleotide (shati-AS), which inhibits the expression of shati mRNA, enhanced the methamphetamine-induced hyperlocomotion, sensitization, and conditioned place preference. Further, blockage of shati mRNA by shati-AS potentiated the methamphetamine-induced increase of dopamine overflow and the methamphetamine-induced decrease in dopamine uptake in the nucleus accumbens. Interestingly, treatment with shati-AS also inhibited expression of TNF-α. Transfection of the vector containing shati cDNA into PC12 cells, dramatically induced the expression of shati and TNF-α mRNA, accelerated dopamine uptake, and inhibited the methamphetamine-induced decrease in dopamine uptake. These effects were blocked by neutralizing TNF-α. These results suggest that the functional roles of shati in methamphetamine-induced behavioral changes are mediated through the induction of TNF-α expression which inhibits the methamphetamine-induced increase of dopamine overflow and decrease in dopamine uptake.

## INTRODUCTION

Drug addiction/dependence is defined as a chronically relapsing disorder that is characterized by compulsive drug taking, inability to limit the intake, and intense drug craving. The positive reinforcing/rewarding effects of drugs of abuse depend mainly on the mesocorticolimbic dopamine system innervating the nucleus accumbens. It is widely accepted that chronic intake of drugs of abuse causes stable changes in the structure and function of the brain, which may be associated with development of drug dependence as well as the long-lived vulnerability to relapse. Using complementary DNA (cDNA) microarrays, changes in the messenger RNA (mRNA) expression profile in relevant brain regions (e.g., nucleus accumbens) have been assessed following chronic administration of abused drugs [1-3]. Evidence from this line of research has implicated nuclear factor-κB (NF-κB) [4] and ΔFosB [5] in signal transduction pathways that modulate behavioral effects induced by drugs and contribute to long-term neuronal changes associated with dependence [6]. In order to elucidate the mechanism, caused by chronic drug abuse, of stable changes in the brain which play a role in the long-lasting behavioral abnormalities of dependent subjects, the candidates for drug-dependence-related genes whose expression was altered by repeated administration of methamphetamine or morphine were screened by using cDNA microarray. We have found that tumor necrosis factor-α (TNF-α) and tissue plasminogen activator (tPA). Since there are many studies that cytokines/neurotrophic factors and extracellular matrix/proteases play critical role in activity-dependent synaptic plasticity and remodeling of the mesocorticolimbic dopaminergic system [7, 8], we focused these molecules. TNF-α plays a neuroprotective role in methamphetamine-induced dependence and neurotoxicity [9] and reduces morphine-induced rewarding effects and behavioral sensitization [10]. Furthermore, the rewarding effects and sensitization induced by methamphetamine and morphine are attenuated by Leu-Ile, an inducer of TNF-α and glial cell line-derived neurotrophic factor (GDNF) [10-14]. On the contrary, the tPA-plasmin system potentiates the rewarding and locomotor-stimulating effects of methamphetamine, morphine, and nicotine by increasing release of dopamine [15-17]. However, the exact neuronal circuits and molecular cascade essential for drug dependence remain unclear. Therefore, we attempt to explore the novel molecules which play more critical role in drug dependence, since the functions of molecules targeted by DNA microarray screening have been already well known.

Recently, we identified a novel molecule ‘shati’ from the NAc of mice treated with methamphetamine using the polymerase chain reaction (PCR)-select cDNA substraction method, which is a differential and epochal cloning technique [18]. In this article, roles of a novel molecule ‘shati’ in the development of methamphetamine-induced dependence are discussed.

## PSYCHOSTIMULANT-INDUCED DEPENDENCE

Drugs of abuse, including methamphetamine, modulate the activity of mesolimbic dopaminergic neurons, projecting from the ventral tegmental area to the nucleus accumbens [19-21]. The psychostimulantory effects of methamphetamine are associated with an increase in extracellular dopamine levels in the brain, *via* facilitating the release of dopamine from presynaptic nerve terminals and inhibition of its reuptake through dopamine transporter [22-24]. In rodent, augmentation of behavioral responses to psychostimulants is observed during and after their repeated administration. Therefore, it has been proposed that activity-dependent synaptic plasticity and remodeling of the mesolimbic dopaminergic system may play a crucial role in drug dependence [25, 26].

## IDENTIFICATION OF SHATI

Since we have preliminarily detected the genes affected by methamphetamine treatment in the nucleus accumbens of mouse using the PCR-select cDNA subtraction method, we pursued shati for intensive investigation: After administration of methamphetamine (2 mg/kg,* s.c.*) or saline for 6 days, an increase of shati mRNA production in the nucleus accumbens was found by 640% in methamphetamine-treated mice with robust behavioral sensitization compared with saline-treated mice. The sequence of cDNA was completely matched to NM_001001985 of NCBI gene bank (the gene record was replaced by NM_001001985.2 on 10-APR-2005). The sequence has been identified by the Mammalian Gene Collection Program Team [27]. Blackshaw *et al.* [28] have demonstrated the extended cDNA sequence by serial analysis of gene expression (SAGE) methods, which provides an unbiased and nearly comprehensive readout of gene expression, and that the gene was for one of the proteins related to the retina development. We have named this novel molecule ‘shati’ after the symbol at Nagoya castle in Japan [18]. The sequence is translated to a protein LOC269642 (protein ID is NP_001001985.1 and 2; 001001985.1 was a part of 001001985.2.).

## CHARACTERIZATION OF SHATI

A motif analysis revealed shati is containing the sequence of GCN5-related *N*-acetyltransferase. Docking simulations with acetyl-CoA or ATP were conducted using Molecular Operating Environment software to calculate the interactive potential energy. In addition, shati contains an acetyl-CoA-binding or ATP-binding site, because the analysis showed the lowest interactive potential energy with acetyl-CoA or ATP. Docking simulations of shati with dopamine, the DNA-binding site, and nuclear localization signals showed very high interactive potential energy or no domain. RT-PCR analysis revealed that shati is expressed at high levels in the cerebrum, cerebellum, liver, kidney, and spleen [18].

## ROLES OF SHATI IN METHAMPHETAMINE-INDUCED HYPERLOCOMOTION, SENSITIZATION, AND CONDITIONED PLACE PREFERENCE

Repeated methamphetamine treatment induces expression of shati mRNA dose-dependently. The expression of shati significantly increased 2, 6, and 24 h after the last methamphetamine treatment and then returned to the control value 1 week after the treatment. Single methamphetamine treatment remarkably induced the expression of shati mRNA in the nucleus accumbens and hippocampus. A methamphetamine or saline challenge on day 6 after repeated administration of methamphetamine for 5 days remarkably induced the expression of shati in the frontal cortex, nucleus accumbens, and caudate-putamen. The increase caused by methamphetamine in the nucleus accumbens was inhibited by pretreatment with either the dopamine D1 receptor antagonist R(+)-SCH23390 or the dopamine D2 receptor antagonist raclopride. Shati is expressed in neuronal cells, but not astro glial cells, of the mouse brain. Treatment with a shati antisense oligonucleotide (shati-AS), which significantly inhibits the expression of shati mRNA, enhanced methamphetamine-induced hyperlocomotion, sensitization, and conditioned place preference. Blockage of shati mRNA by shati-AS potentiated the methamphetamine-induced increase of dopamine overflow in the nucleus accumbens and the methamphetamine-induced decrease in synaptosomal and vesicular dopamine uptake in the midbrain. These results suggest that ‘shati’ is involved in the development of methamphetamine-induced hyperlocomotion, sensitization, and conditioned place preference. The functional roles of shati in methamphetamine-induced behavioral alterations are likely to be mediated by its inhibitory effects on the methamphetamine-induced increase of dopamine overflow in the nucleus accumbens and on the methamphetamine-induced decrease in dopamine uptake in the midbrain [18].

## ROLES OF SHATI ON DOPAMINE UPTAKE – THE RELATIONSHIP SHATI AND TNF-α

**Fig. (1):**
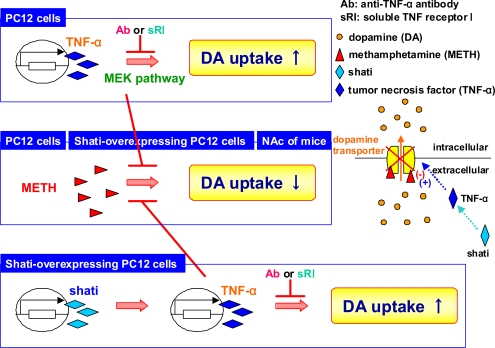
**Roles of shati on dopamine uptake – the relationship shati and TNF-α.** TNF-α increased dopamine uptake *via* the mitogen-activated protein kinase kinase (MEK) signaling pathway in PC12 cells. The increase was antagonized by the anti-TNF-α antibody (Ab) and soluble TNF receptor I (sRI), suggesting that TNF-α certainly increases dopamine uptake in PC12 cells. Moreover, TNF-α inhibited the methamphetamine-induced decrease in dopamine uptake in PC12 cells. Overexpression of shati increased dopamine uptake and inhibited the methamphetamine-induced decrease in dopamine uptake in PC12 cells by increasing TNF-α expression, since these effects were antagonized by anti-TNF-α antibody and soluble TNF receptor I.

**Fig. (2):**
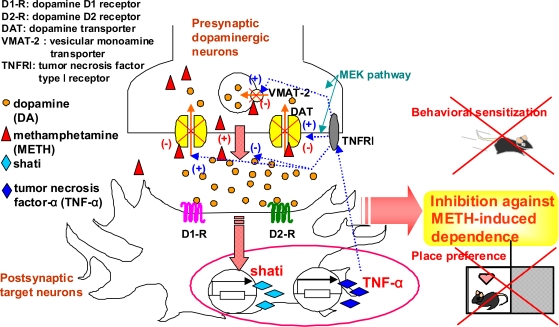
**The functional roles of shati in the development of methamphetamine-induced dependence.** Under basal conditions, plasmalemmal dopamine transporter is involved in the reuptake of extracellular dopamine into the cytosol; subsequently the cytosolic dopamine is stored into synaptic vesicles *via* vesicular monoamine transporter-2. Treatment of methamphetamine inhibits dopamine uptake through dopamine transporter and facilitates dopamine’s release from presynaptic nerve terminals, resulting in potentiation of the methamphetamine-induced dependence. Methamphetamine induces shati and TNF-α expression in the target neurons through the activation of dopamine receptors. TNF-α induced by shati inhibits the methamphetamine-induced increase of dopamine overflow in the nucleus accumbens by promoting dopamine uptake *via* mitogen-activated protein kinase kinase (MEK) pathway and finally inhibits sensitization to and the rewarding effects of methamphetamine. Targeting the shati-TNF-α system would provide a new therapeutic approach to the treatment of methamphetamine dependence.

Interestingly, treatment with shati-AS also inhibits expression of TNF-α *in vivo*. To examine the relationship between shati and TNF-α, and the precise mechanism behind the role of shati in dopamine uptake and the methamphetamine-induced decrease in dopamine uptake, we established a PC12 cell line which is transfected the vector containing shati cDNA. TNF-α increased dopamine uptake *via* the mitogen-activated protein kinase pathway, and inhibited the methamphetamine-induced decrease in dopamine uptake in PC12 cells (Fig. **1**). Shati was expressed in TNF-α-immunopositive cells. Transfection of the vector containing shati cDNA into PC12 cells, dramatically induced the expression of shati and TNF-α mRNA, accelerated dopamine uptake, and inhibited the methamphetamine-induced decrease in dopamine uptake (Fig. **1**). These effects were blocked by neutralizing TNF-α (Fig. **1**). These results suggest that the functional roles of shati in methamphetamine-induced behavioral changes are mediated through the induction of TNF-α expression which inhibits the methamphetamine-induced increase of dopamine overflow and decrease in dopamine uptake [29] (Figs. **1** and **2**). Targeting the shati-TNF-α system would provide a new therapeutic approach to the treatment of methamphetamine dependence [18, 29] (Fig. **2**).

## DISCUSSION

As reviewed in this article, the roles of ‘shati’, which have been identified as a specific candidate molecule for the development of methamphetamine-induced dependence, were discussed. Recent evidence has demonstrated that various cytokines and proteinases also participate to the development and relapse of drug dependence, which may be divided into two groups. Anti-addictive factors such as shati [18, 29], piccolo [30], TNF-α [9, 10], and GDNF [8, 11-14, 31] act to reduce the rewarding effect of drugs of abuse. Pro-addictive factors that act to potentiate the rewarding effect of drugs include basic fibroblast growth factor (bFGF) [32], brain-derived neurotrophic factor (BDNF) [7, 33], tPA [15-17], matrix metalloproteinase (MMP)-2 and MMP-9 [34, 35]. These findings suggest that an imbalance between anti-addictive and pro-addictive factors contributes to the development and relapse of drug dependence [14, 26]. We propose that the dynamic changes in and balance of levels of anti-addictive and pro-addictive factors in the brain are one of the determinants of susceptibility to drug dependence.
